# Rare Presentation of Disseminated Gout Nodulosis and Chronic Inflammatory Arthritis

**DOI:** 10.1155/2023/8083212

**Published:** 2023-06-14

**Authors:** Faria Sami, Shahzad Ahmed Sami, Shilpa Arora

**Affiliations:** ^1^John H. Stroger Jr. Hospital of Cook County, 1969 W Ogden Ave, Chicago, IL 60612, USA; ^2^Trinity Health Oakland Campus, 44405 Woodward Ave, Pontiac, MI 48341, USA

## Abstract

**Background:**

Gout is an inflammatory arthritis caused by monosodium urate (MSU) deposition. Acute gout is a dramatic painful swelling of the joint; however, MSU can deposit in other tissues as well, including skin, gastrointestinal tract, and bones over time. Disseminated tophi in the skin are a rare presentation of gout known as gout nodulosis. We present a case of gout nodulosis with subcutaneous diffuse miliary nodules in nonarticular areas with concurrent findings suggestive of chronic inflammatory arthritis. *Case Presentation*. A 39-year-old patient presented with intermittent painful swelling in multiple joints with prolonged morning stiffness. On exam, synovitis was present in multiple proximal interphalangeal joints, wrists, elbows, and knees. Chronic raised pearly nodular rash and swellings on extensor aspects of arms, legs, and anterior abdomen were noticeable. He had negative rheumatoid factor and anti-CCP antibody, C-reactive protein of 0.23 mg/dL, erythrocyte sedimentation rate of 37 mm/hr, and uric acid of 10.6 mg/dL. Hand X-rays revealed severe periarticular osteopenia and joint space narrowing in several joints. Musculoskeletal ultrasound showed a double contour sign at multiple joints and a tophaceous deposit over the olecranon fossa. The biopsy of the nodular rash was consistent with tophi. He was diagnosed with chronic tophaceous gout with skin nodulosis and possible overlap of seronegative rheumatoid arthritis given his X-ray findings.

**Conclusion:**

This case discusses one of the rare presentations of gout with disseminated gouty tophi in the skin to raise clinical awareness. The clinical dilemma of the overlap of gout and rheumatoid arthritis posing a diagnostic challenge for clinicians is also highlighted.

## 1. Introduction

Gout is an inflammatory arthritis caused by deposition of monosodium urate (MSU) crystals in the joints. Risk factors are divided into nonmodifiable such as advanced age, male gender, ethnicity, and genetic factors and modifiable including obesity, alcohol use, medications, diuretics, chronic renal failure, and high purine diet [1, 2]. Chronic gout is characterized by deposition of urate crystals in soft tissues and skeleton known as tophi. Tophi can be present in places like skin, gastrointestinal tract, bones, synovium, tendons, ligaments, and the nerves [3–5]. Tophi in general and nonarticular atypical presentations of tophi are associated with higher morbidity and deformities including joint destruction, bony erosions, skin ulceration, and even, rarely, panniculitis and lipohypertrophy [6].

We discuss a case of rare presentation of gout with disseminated subcutaneous tophaceous nodules and chronic inflammatory arthritis and review existing literature.

### 1.1. Case Report

**Figure 1:**
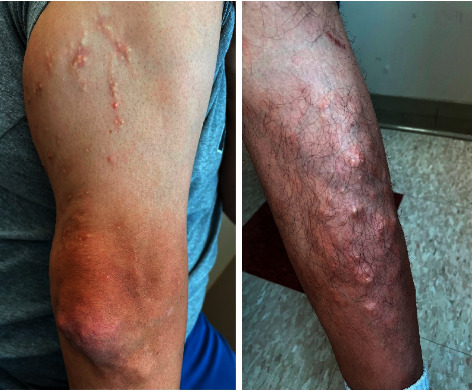
Nodular rash on extensor surfaces of upper extremity lower extremity.

**Figure 2:**
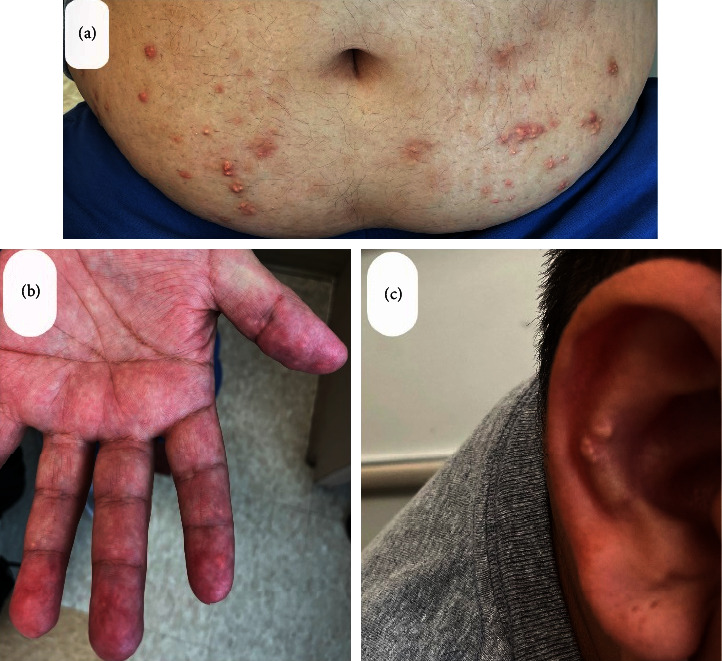
Pearly white nodular rash on anterior abdomen (a) fingertips (b), and auricle (c).

A 39-year-old Hispanic male with no known medical history presented with progressive pain and swelling in multiple bilateral hand joints, elbows, and knees for the last 5 years with intermittent flare ups, associated with prolonged morning stiffness. He had difficulty making a fist and ambulating. He denied any chronic medications, red meat, or seafood intake but consumed 4 beers/day few days a week for last several years. On exam, synovitis was present in bilateral several proximal interphalangeal (PIP) joints, wrists, elbows, and knees. Range of motion was severely restricted in bilateral wrists with poor fist formation. He had nontender raised pearly nodular rashes on extensor aspects of arms, legs, and anterior abdomen for more than a year (Figures 1 and 2). Large subcutaneous swellings were noted on extensor surfaces of both elbows and anterior shins.

**Figure 3:**
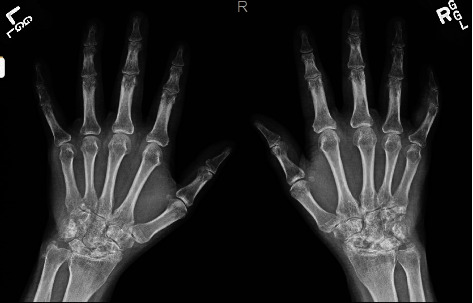
Bilateral hand X-rays with diffuse periarticular osteopenia, diffuse severe joint space narrowing involving carpel bones and radiocarpal joints, erosive changes in bilateral ulnar styloid processes, 4th and 5th carpometacarpal joints, and radiocarpal and radioulnar joints.

Labs showed hemoglobin of 10 g/dL (normal 12–16), negative antinuclear antibody, rheumatoid factor, anti-CCP antibody, C-reactive protein of 0.23 mg/dL (normal 0–0.5), erythrocyte sedimentation rate of 37 mm/hr (normal 0–20), and uric acid of 10.6 mg/dL (normal 3–7). Bilateral hand X-rays (Figure 3) revealed severe periarticular osteopenia, severe joint space narrowing at radiocarpal, intercarpal, and carpometacarpal joints along with erosions in carpal bones and ulnar styloid processes. Musculoskeletal ultrasound showed double contour sign at multiple joints and tophaceous deposit over the right olecranon fossa. Nodular rash biopsied by dermatology was consistent with tophi on pathology. He was diagnosed with chronic tophaceous gout with nodulosis with overlap of seronegative rheumatoid arthritis (RA) given exam findings of symmetric wrist arthritis with severe reduced range of motion and X-ray findings suggestive of RA. He was started on urate-lowering therapy with allopurinol 100 mg daily, colchicine 0.6 mg BID for gout, and low-dose steroids (prednisone 10 mg daily) for RA. He had elevated liver enzymes at baseline and was also found to have latent tuberculosis, hence, additional DMARDs were not initiated at first. He was started on hydroxychloroquine and methotrexate eventually with tapering of steroids and clinical improvement in his symptoms along with exam findings. Allopurinol is being up titrated to target serum uric acid of less than 5.

## 2. Discussion

Gout attacks can be monoarticular or polyarticular and present with swelling, warmth, and tenderness of the joint sometimes with fever and malaise. Polyarticular arthritis is more common with chronic tophaceous gout [7]. Chronic tophaceous gout develops in approximately 10 years in untreated patients, and 12–35% develops tophi [8, 9]. Diagnosis is usually clinical, but synovial fluid analysis for monosodium urate crystal is gold standard. Tophaceous gout can form soft tissue calcifications anywhere with predisposition for area of prior trauma [10, 11]. The double contour sign on ultrasound is suggestive of MSU deposition. The presence of double contour sign on ultrasound and age are risk factors for tophi [12].

The tophi can mimic several other nodules including Heberden's nodes, rheumatoid nodules, and even malignancy [13]. The prevalence of tophi can range 3%–21% in gout patients and hyperuricemia is an independent risk factor for it [14, 15]. Disseminated subcutaneous deposition is referred to as gout nodulosis or miliarial gout, an extremely rare manifestation. These are “milia-like” nodules of white-creamy tophi deposited in the skin with underlying erythema, which can easily be mistaken as acne or folliculitis [16, 17]. Few case reports discuss that nodulosis can be present without gout as well as an initial presentation of gout and is usually associated with hyperuricemia [18, 19]. Although the exact pathogenesis of severe intradermal involvement is unknown, some risk factors include obesity, chronic venous insufficiency, and prolonged use of corticosteroids [17]. Shukla et al. further discuss renal insufficiency, chronic diuretic use, and hypertension as potential risk factors for intradermal tophi [20]. Clinical implications of severe skin involvement by tophaceous gout include multiple skin ulcerations, panniculitis, and skin/soft tissue infections. At later stages, these could result in irreversible skin disfigurement and joint destruction [21]. Besides skin biopsy, dual energy computed tomography scan (DECT) can be a useful tool to detect MSU crystals within the skin lesions.

Our patient presented with chronic pain and swelling of several small and large joints suggestive of chronic inflammatory arthritis. Musculoskeletal ultrasound confirmed MSU deposition. Given the atypical appearance of subcutaneous nodules, skin biopsy was pursued which was consistent with tophi (MSU deposition). His chronic polyarticular arthritis was suspected to be from chronic tophaceous gout arthropathy versus RA at first. He had clinical synovitis on exam. Serologies for RA were negative, but the characteristic findings of periarticular osteopenia in several joints and severe joint space narrowing in the bilateral intercarpal and radiocarpal joints along with erosions on X-rays supported the diagnosis of rheumatoid arthritis overlap. He has also responded remarkably to methotrexate and hydroxychloroquine the standard conventional drugs for RA.

RA and gout overlap is uncommon. One longitudinal study reports gout in 6.1% and hyperuricemia >6.8 mg/dL in 17% of RA patients; however, presence of neither of these was associated with severity of RA disease activity [22]. It is often a diagnostic challenge with overlapping features of the two. A review of literature of this overlap reports that 75% of the patients had gout before the diagnosis of rheumatoid arthritis, which was largely negative for RF and anti-CCP antibody when copresent with gout [23]. Another large population-based study comparing RA patients with healthy controls reported a significantly high odds of gout in RA patients [24]. The presence of MSU crystals in RA patients is also noted to be significantly high in one study, with about 70% seronegative RA patients demonstrating MSU crystals DECT scan.

We present a rare case of the Gout nodulosis with chronic inflammatory polyarthritis to raise awareness about atypical presentations of gout and discuss the conundrum of coexisting rheumatoid arthritis.

## Data Availability

The data used to support the findings of this study are included within the article.
